# Clinical Characteristics Associated with Suicide Attempts in Clinical Settings: A Comparison of Suicidal and Non-Suicidal Depressed Inpatients

**DOI:** 10.3389/fpsyt.2016.00109

**Published:** 2016-06-20

**Authors:** Carla Gramaglia, Alessandro Feggi, Paola Bergamasco, Fabrizio Bert, Eleonora Gattoni, Debora Marangon, Roberta Siliquini, Eugenio Torre, Patrizia Zeppegno

**Affiliations:** ^1^Department of Translational Medicine, Institute of Psychiatry, Università del Piemonte Orientale, Novara, Italy; ^2^SC. Psichiatria, AOU Maggiore della Carità di Novara, Novara, Italy; ^3^Department of Public Health and Paediatric Sciences, Università degli Studi di Torino, Torino, Italy

**Keywords:** suicide attempt, depression, clinical management, antidepressants, inpatient

## Abstract

**Introduction:**

Both psychiatrists and psychiatric nurses are involved in the psychiatric management of suicidal inpatients. One-to-one observation by qualified nurses and the accommodation of the patient in a room close to the infirmary are usually recommended. Suicidal risk should be reassessed periodically to check response to treatment.

**Aim:**

To compare the severity of depressive symptoms in depressed inpatients admitted after an attempted suicide and those admitted for any other reason and to assess the severity of suicide attempts and the management of suicidal risk in clinical settings.

**Materials and methods:**

We divided the sample into two subgroups: patients with a diagnosis of depression admitted because of a recent suicide attempt and depressed patients with no recent history of attempted suicide. Socio-demographic and clinical data were gathered; assessments included the Montgomery-Asberg Depression Rating Scale and the Nurses’ Global Assessment of Suicide Risk (NGASR).

**Results:**

Forty-six patients were recruited over a 1-year period: 20 were admitted to the hospital following a suicide attempt; the other 26 had not attempted suicide and were admitted for other depression-related reasons. Multivariate analysis revealed a correlation between use of antidepressants and recent attempted suicide. Attempting suicide was not related to the severity of depressive symptoms. In the recent suicide attempt subgroup, NGASR suicide risk levels were lower at discharge than at admission. Patients with a recent history of attempted suicide had a higher number of suicide attempts in their clinical history than patients with no recent history of attempted suicide.

**Conclusion:**

There were no correlations between psychiatric diagnosis, severity of depressive symptoms, and recent suicide attempt. Antidepressant therapy protected against suicide attempts. History of suicide attempts was one of the best predictors of recent attempted suicide. A more thorough understanding of the complex phenomenon of suicide and the reasons for suicidal behavior is needed.

## Introduction

Suicide is a significant public health problem: more than 800,000 people (11.4/100000) die by suicide every year according to the World Health Organisation (1) and in 2012 suicide was the 15th cause of death worldwide. Attempted suicide is far more common than suicide (2, 3); the prevalence ranges from 0.3% in high income countries to 0.4% in lower income countries (2).

A suicide attempt often leads to admission to a psychiatric ward and it represents a challenge for the whole clinical team. Suicide attempts after hospitalization are also a major cause of morbidity. Arguably risk of attempted or successful suicide is highest at the time of hospitalization (4). Predicting suicide during psychiatric hospitalization remains a challenge (5). Large and Ryan recently claimed that, although it is common to repeat assessments of suicide risk during a hospital stay, this poses several problems of interpretation, and that the predictive value of the risk categories assessed is inevitably low (6). The risk factors most reliably associated with inpatient suicide are “static” ones, and include a diagnosis of affective disorder, a history of suicide attempts, and a suicide attempt in the week before psychiatric admission (7, 8). Although the identification of suicide risk factors does not appear to contribute to a useful probabilistic estimate of inpatient suicide risk, one would expect that some suicides could be prevented by addressing them (6).

According to the 2003 guidelines of the American Psychiatric Association (9), the starting point for the psychiatric management of patients who exhibit suicidal behavior is the establishment and maintenance of a therapeutic alliance involving psychiatrists and psychiatric nurses working in cooperation with the patient’s general practitioner, mental health service psychiatrists, and family members or caregivers. Specific precautions are required to ensure patient safety although excessive restraint should be avoided. These might include one-to-one observations by qualified nurses if the risk is severe, and accommodating the patient in a room close to the infirmary is usually recommended. The importance of appropriate clinical management is supported by evidence that inpatient suicide attempts are more likely to occur during shift changes or when staff are less alert (10). Assessment and management of patients who have attempted suicide is complex, and the limited number of effective approaches represents an important problem (11). There is evidence supporting the use of antidepressants, lithium, and other mood stabilizers to reduce symptom-related suicide risk. It is important not to ignore the fact that suicidal behaviors may occur independently from a diagnosis of depression or, in wider terms, any psychiatric diagnosis (12–14). This is an important issue, considering the current zeitgeist and the growing tendency to sue psychiatrists after a patient’s suicide. The approach to suicide and suicide attempt should not overlook the philosophical and existential perspective. According to Camus, “There is but one truly serious philosophical problem and that is suicide. Judging whether life is or is not worth living amounts to answering the fundamental question of philosophy. All the rest – whether or not the world has three dimensions, whether the mind has nine or twelve categories – comes afterwards. These are games; one must first answer” (15).

On the basis of this evidence, the aim of our study was to compare socio-demographic, clinical, and treatment variables in depressed inpatients admitted to our psychiatry ward following a suicide attempt and those admitted for any other reason. The clinical implications of our findings are discussed.

## Materials and Methods

We collected data from all depressed inpatients admitted to the psychiatry ward, A.O.U. “Maggiore della Carità” Novara between November 30, 2014 and November 30, 2015. Inclusion criteria were as follows: diagnosis of depression, age >18 years, adequate understanding of Italian, and willingness to provide written consent to participation. Mental retardation and dementia were exclusion criteria. We divided the sample into two subgroups: patients with a diagnosis of depression admitted due to a recent suicide attempt (recent suicide attempters; RSAs) and patients with a diagnosis of depression who had been admitted for a reason other than attempted suicide (non-suicide attempters; NSAs).

Socio-demographic (age and gender), clinical, and treatment-related information was gathered, including lifetime diagnoses according to DSM-5 criteria (16), comorbid physical illness, suicidal behavior, medication during hospital stay, and length of stay (LOS). Data were gathered using clinical interviews and clinician- and nurse-rated scales. The Montgomery-Asberg Depression Rating Scale (MADRS) (17), a 10-item semi-structured scale, was used to assess the presence and severity of depressive symptoms. MADRS scores are classified as follows: 0–12, minimal depression; 13–19, mild depression; 20–34, moderate depression; ≥35, severe depression. Inter-item reliability for the MADRS is 0.86 (18).

The Nurses’ Global Assessment of Suicide Risk (NGASR) was used to evaluate and measure suicidal risk in RSAs during hospitalization (19). The Italian version of the NGASR was filled in by trained nurses for RSAs whenever a change in behavior was observed. Total NGASR score indicates level of suicidal risk: ≤4, low risk (standard daily nursing care; test re-evaluation if needed); 5–8, intermediate risk (standard nursing care three times a day; re-evaluation every 72 h); 9–11, high risk (standard nurse care every 15 min throughout the day and night; re-evaluation every 24 h); ≥12, very high risk (constant, dedicated nursing care or other professional care; re-evaluation every 24 h).

This research project was approved by the Institutional Review Board of the Università del Piemonte Orientale as part of the research duties of the Psychiatry Institute. Informed consent was obtained from all participating patients.

### Statistical Analysis

Descriptive statistics, frequencies and percentages for categorical variables and means and SDs for continuous variables were calculated. Group differences were evaluated with the chi-squared test (categorical variables) or *t*-test (continuous variables). After this, multivariate logistic regression was performed to assess the potential predictors of suicide attempts. The covariates included in the final model were selected using the Hosmer and Lemeshow procedure, by inserting variables with a univariate *p*-value <0.25 as the main criterion (20). Results are expressed as odds ratios (ORs) with 95% confidence intervals (95%CIs). The statistical significance level was set at *p* ≤ 0.05. Statistical analyses were performed with STATA 11 (21).

## Results

During the 1-year period described above, 326 patients were admitted to our psychiatry ward, 46 (23.91% men; 76.09% women) received a diagnosis of depression and were, thus, eligible for our study. Twenty of them (25% men; 75% women) had been admitted to the hospital after attempting suicide, while the other 26 (23.08% men; 76.92% men) had not attempted suicide and were admitted for other reasons related to their depression. The mean age for the whole sample was 49.78 ± 15.81 years (RSA: 49.69 ± 16.00 years; NSA: 49.90 ± 15.98 years). Clinical variables, treatment, and MADRS scores for both groups are reported in Table 1.

**Table 1:** **Group comparison of clinical and treatment variables and MADRS scores**.

Variable		RSA (*n* = 20) %, *n*	NSA (*n* = 26) %, *n*	*P*
Lifetime diagnosis	Bipolar disorder I	0.00 (0)	3.85 (1)	0.278
	Bipolar disorder II	10.00 (2)	19.23 (5)	
	Major depressive disorder	70.00 (14)	42.31 (11)	
	Dysthymia	20.00 (4)	34.62 (9)	
Psychiatric comorbidity	Yes	40.00 (8)	34.62 (9)	0.708
	No	60.00 (12)	65.38 (17)	
Physical comorbidity	Yes	50.00 (10)	57.69 (15)	0.604
	No	50.00 (10)	42.31 (11)	
Medication	Benzodiazepines	65.00 (13)	80.77 (21)	0.227
	Mood stabilizers	10.00 (2)	23.08 (6)	0.246
	Antipsychotic	40.00 (8)	42.31 (11)	0.875
	Antidepressants	65.00 (13)	84.62 (22)	0.122
Previous suicide attempts	0	30.00 (6)	76.92 (20)	**<0.001**
	1	35.00 (7)	11.54 (3)	
	2	30.00 (6)	11.54 (3)	
	>2	5.00 (1)	0.00 (0)	
MADRS	Minimal symptoms	15.00 (3)	7.69 (2)	0.645
	Mild depression	35.00 (7)	34.62 (9)	
	Moderate depression	40.00 (8)	53.85 (14)	
	Severe depression	10.00 (2)	3.85 (1)	

*Bold font means statistically significant results *p* ≤ 0.05*.

Psychiatric comorbidities in the RSA group included obsessive compulsive disorder (*n* = 1), substance-related and addictive disorders (*n* = 1), and personality disorders (borderline personality disorders, *n* = 2; narcissistic personality disorder, *n* = 3; histrionic personality disorder, *n* = 1). In the NSA group, psychiatric comorbidities included mental retardation (*n* = 1), substance-related and addictive disorders (*n* = 2), and personality disorders (borderline personality disorders, *n* = 1; histrionic personality disorder, *n* = 3; obsessive personality disorder, *n* = 1; personality disorder not otherwise specified, *n* = 1). We also assessed comorbid cardiovascular, endocrinological, gastrointestinal, neurological and urologic diseases, cancer, and others.

Mean LOS was 14 ± 9.9 days for the RSA group and 14 ± 6.5 days for the NSA group.

The methods used in recent suicide attempts by the RSA group were drug poisoning (65%; *n* = 13), cutting (20%; *n* = 4), or other methods (drowning, jumping from heights and use of firearms; 15%; *n* = 3). In the RSA group, the distribution of suicide risk on admission based on NGASR scores was as follows: low risk: 10% (*n* = 2); moderate risk: 35% (*n* = 7); high risk: 30% (*n* = 6); very high risk: 25% (*n* = 5). At discharge, the corresponding figures were 65% (*n* = 13), 25% (*n* = 5), 10% (*n* = 2), and 0% (*n* = 0).

Table 2 shows the results of the multivariate analysis for the assessment of potential risk factors related to suicide attempt.

**Table 2:** **Predictors of suicide attempt**.

Variable		Adjusted OR	95% CI	*p*
Age		1.01	(0.95–1.09)	0.611
Gender	Female	1.28	(0.17–9.55)	0.811
Psychiatric comorbidity	Yes	0.19	(0.02–1.61)	0.129
Physical comorbidity	Yes	0.36	(0.04–3.06)	0.346
Therapy	Benzodiazepines	1.09	(0.11–10.70)	0.938
	Mood stabilizers	1.23	(0.09–17.68)	0.880
	Antipsychotic	0.12	(0.01–1.44)	0.095
	Antidepressants	0.03	(0.01–0.49)	**0.013**
Previous suicide attempts	0	1		–
	1	88.34	(3.79–2061.28)	**0.005**
	2	53.98	(342–851.98)	**0.005**
	>2	–		–

*Bold font means statistically significant results *p* ≤ 0.05*.

## Discussion

Aside from previous suicide attempts, psychopathology is considered one of the most important predictors of suicidal behavior (22, 23). Major depressive episodes associated with major depressive disorder or bipolar disorder account for at least half of all suicide deaths (24). A recent meta-analysis concluded that despite the heterogeneity of relevant studies, there is a stronger association between suicidal behavior and schizophrenia and other psychotic disorders than between suicidal behavior and mood disorders (25).

Patients with physical illnesses, such as chronic obstructive pulmonary disease, cardiovascular diseases, osteoporosis, multiple sclerosis, inflammatory bowel disease, migraine, epilepsy, and traumatic brain injuries, are also at higher risk of suicide than the healthy population (26). In our sample, however, there were no differences between the RSA and NSA groups with respect to physical comorbidities and we also found no correlations between lifetime psychiatric diagnosis (bipolar I, bipolar II, major depressive disorder, dysthymia), psychiatric and physical comorbidity, and suicidal behavior.

Recent suicide attempt was not related to the severity of depressive symptoms as measured with the MADRS (*p* = 0.645). Although the small sample size should not be overlooked when interpreting our results, this nevertheless suggests that suicidal behaviors in depressed patients are linked to factors other than the severity of depressive symptoms. A recent study reported that increased risk of suicide attempts in mood disorders was associated with impulsivity and unstable relationships (27). This is consistent with clinical experience which suggests that suicide attempts may be influenced by many other factors in addition to psychiatric diagnosis and severity of psychiatric symptoms, e.g., personality, resilience, personal and cultural factors, and beliefs (12–14).

There were no group differences in use of any of the classes of medication analyzed (benzodiazepines, antidepressants, antipsychotics, mood stabilizers). This is not surprising, given that there is only empirical evidence to support the use of a very limited number of drugs to treat non-suicidal self-injurious behavior (28, 29). This suggests that the RSA and NSA groups would receive similar treatment and, in fact, there was no group difference in LOS.

The only clinical difference between the groups was in number of previous suicide attempts. The number of suicide attempts in the clinical history was higher in the RSA group. This result is consistent with the large body of evidence showing that a history of attempted suicide is a strong risk factor for subsequent suicidal behavior (7, 8, 30–32). Multivariate analysis showed that a history of one (OR = 88.34, *p* = 0.005) or two (OR = 53.98, *p* = 0.005) previous suicide attempts was a strong predictor of current suicidal behavior. There are reports suggesting that 16–34% of people who attempt suicide try again within 1 or 2 years after their first attempt and that there is a history of previous non-fatal suicide attempts in up to 40% of fatal suicides (33). Hence, a history of suicidal behavior, independent of psychiatric diagnosis, is likely to be the strongest risk factor for successful suicide.

Multivariate analysis indicated that, in the RSA group, use of antidepressants was protective against recent suicide attempt (adjusted OR = 0.03, 95% CI: 0.01–0.49), although we should not overlook the fact that the reference category for this analysis was patients not using any psychopharmacological therapy. Evidence on the relationship between use of antidepressants and suicide risk is mixed; although there is broad agreement that selective serotonin reuptake inhibitors (SSRIs) do not increase suicide risk (34), there is no evidence that antidepressants have a specific protective effect with respect to suicidal behavior. For instance, a recent retrospective cohort study comparing depressed patients treated with antidepressants and untreated patients found no difference in suicide rate between the two groups (35). The small number of drugs for which there is empirical evidence of a reduction in non-suicidal self-injurious behavior includes some SSRIs and venlafaxine (36).

Consistent with the literature, patients in our sample with a history of suicide attempts were at greater risk of a further attempt than those without such history and, hence, from a clinical standpoint, behavioral monitoring of this group during hospitalization is essential. Suicidal risk assessment by clinicians and nurses may be helpful in tailoring treatment and assistance to the patient’s needs (7, 8, 37, 38).

The literature suggests that patients who have recently attempted suicide should be managed by experienced nurses because suicide and suicidal risk assessment are multifaceted, complex phenomena (39). In our clinical practice, the NGSAR has proved to be useful for assessing suicidal risk and monitoring inpatients. There is evidence that in adult patients suffering from major mood disorders, suicide risk remains high in the hours following a suicide attempt and admission to the emergency department (40) and also in the days following admission to a psychiatric ward (41). Nonetheless in the RSA group, which had a mean LOS of 14 ± 9.9 days, we observed a considerable decrease in NGASR scores between admission to the psychiatric ward and discharge.

### Limitations

Some limitations of our study should be underscored. The sample size limits the generalizability of our results, as does the setting, which was limited to a single psychiatric ward. Anyway it should be noted that our ward is part of the Azienda Ospedaliero Universitaria (AOU) Maggiore della Carità Hospital, the second largest hospital in Piedmont, which may be considered representative of the North-Eastern Piedmont area. We also need to point out that the risk of suicide is high in Piedmont, particularly in the north-eastern areas (42–44). The number of variables assessed in this study was limited, and we cannot exclude the possibility that other clinical or socio-demographic factors may predict suicide attempts in depressed patients.

The strength of our research is that we analyzed data from real-life settings. More similar studies are warranted to help and support clinicians in their everyday clinical practice.

## Conclusion

Notwithstanding the limits noted by Large and Ryan (6), the importance of interventions designed to reduce or prevent suicide during inpatient psychiatric treatment has been recently underscored (45–47). There is still scant evidence on this issue and little research into the effectiveness of prevention strategies (48, 49).

Reduction of suicide risk in psychiatric wards may be achieved by creating a safe environment and ensuring patient visibility; patients should be properly supervised and assessed through teamwork and sharing of viewpoints within the team. Clinicians and nurses should be aware of suicide risk and respect it (50); using structured measures to assess suicide risk may be helpful. Withdrawing from ward activity, wanting to leave the ward without permission and non-compliance with medication regimes should be treated as indicators of risk that warrant early intervention (51).

Interestingly, we found no correlations among psychiatric diagnosis, psychiatric and physical comorbidity, severity of depressive symptoms, and suicidal behavior. On the other hand, antidepressant therapy was found to protect against suicide attempts. A more thorough understanding of the complex phenomenon of suicide and the reasons for suicidal behavior is needed. Consideration should be given to the existential perspective as this may help to de-stigmatize suicide and correct the impression that only psychiatric patients commit suicide.

## Author Contributions

CG, ET, and PZ were responsible for the conception and design of the work; data collection was carried out by PB, AF, EG, and DM; data analysis and interpretation by FB and RS; the article was drafted by AF, EG, and CG, and it was revised by PZ. All the authors have approved the final version for publication.

## Conflict of Interest Statement

The authors declare that the research was conducted in the absence of any commercial or financial relationships that could be construed as a potential conflict of interest. The reviewer (AV) declared a past co-authorship with one of the authors (PZ) to the handling Editor, who ensured that the process met the standards of a fair and objective review.

## Abbreviations

MADRS, the Montgomery–Asberg Depression Rating Scale; NGASR, the Nurses’ Global Assessment of Suicide Risk; NSA, non-suicide attempters; SA, suicide attempters.
